# Cardiotonic pill attenuates white matter and hippocampal damage *via* inhibiting microglial activation and downregulating ERK and p38 MAPK signaling in chronic cerebral hypoperfused rat

**DOI:** 10.1186/1472-6882-13-334

**Published:** 2013-11-26

**Authors:** Ki Mo Lee, Ji Hye Bang, Jung-Soo Han, Bu Yeo Kim, In Sun Lee, Hyung Won Kang, Won Kyung Jeon

**Affiliations:** 1 KM-Based Herbal Drug Development Group, Korea Institute of Oriental Medicine, Daejeon 305-811, Republic of Korea; 2 Department of Biological Sciences, Konkuk University, 1 Hwayang-dong, Gwangjin-gu, Seoul 143-701, Republic of Korea; 3 Department of Neuropsychiatry, College of Oriental Medicine, Won-Kwang University, Iksan 570-749, Republic of Korea

**Keywords:** Cardiotonic pill, Permanent bilateral common carotid artery occlusion, Inflammation, Microglia, White matter, Hippocampus

## Abstract

**Background:**

The cardiotonic pill (CP) is a herbal medicine composed of *Salvia miltiorrhiza* (SM), *Panax notoginseng* (PN), and *Dryobalanops aromatica Gaertner* (DAG) that is widely used to treat cardiovascular diseases. The present experiment was conducted to examine the effects of CP on white matter and hippocampal damage induced by chronic cerebral hypoperfusion.

**Methods:**

Chronic cerebral hypoperfusion was induced in male Wistar rats by permanent bilateral common carotid artery occlusion (BCCAo). Daily oral administration of CP (200 mg/kg) began 21 days after BCCAo and continued for 42 days. The levels of microglial activation and myelin basic protein (MBP) were measured in the white matter and hippocampus of rats with chronic BCCAo, and the expression levels of mitogen-activated protein kinases (MAPKs) and inflammatory markers such as cyclooxygenase-2, interleukin-1β, and interleukin-6 were examined.

**Results:**

MBP expression was reduced in the white matter and hippocampal regions of rats that received BCCAo. In contrast, reduced levels of MBP were not observed in BCCAo rats given CP treatments. The administration of CP alleviated microglial activation, the alteration of ERK and p38 MAPK signaling, and inflammatory mediator expression in rats with chronic BCCAo.

**Conclusion:**

These results suggest that CP may have protective effects against chronic BCCAo-induced white matter and hippocampal damage by inhibiting inflammatory processes including microglial activation and proinflammatory mediator expression, and downreguating the hyperphosphorylation of ERK and p38 MAPK signaling.

## Background

A chronic reduction in cerebral blood flow may lead to vascular cognitive impairment [1]. Decreased cerebral blood flow is observed in patients with Alzheimer’s disease (AD) or vascular dementia (VaD) [2,3]. Rats with chronic cerebral hypoperfusion induced by permanent bilateral common carotid artery occlusion (BCCAo) are used to experimentally study pathological mechanisms and to develop therapeutic treatments of cognitive impairment in human vascular disease [4]. Reduced cerebral blood flow occurs immediately in rats after BCCAo and lasts for 2–3 days. Seven days later, a moderate (20-40%) but persistent reduction in cerebral blood flow remains for several months [5]. Rats treated with chronic BCCAo exhibit many features of human VaD and AD [4,5].

One pathological feature in the brains of chronic BCCAo rats is white matter lesions, which are frequently observed in the brains of patients with VaD [6]. Chronic BCCAo rats show cognitive impairments and altered signaling in brain structures, including the hippocampus [4,7]. In addition, it has been reported that neuroinflammation is associated with the development of white matter lesions and hippocampal damage in BCCAo rats [8,9]. For example, microglial activation is observed in the white matter and hippocampus. Microglial activation may aggravate damage to these structures through the excessive production of reactive oxygen species and proinflammatory cytokines as well as through the activation of MAPK signaling [4,10,11].

Herbal medicines have traditionally been used to treat various diseases. Recently, studies have been conducted to reveal the mechanisms of these medicines and to demonstrate the effectiveness of these medicines for other diseases. One of these herbal medicines is the cardiotonic pill (CP), which is composed of *Salvia miltiorrhiza* (SM), *Panax notoginseng* (PN), and *Dryobalanops aromatica Gaertner* (DAG). This pill has been widely used in Korea, China, and Russia for the prevention and management of vascular diseases, such as occlusive vasculitis, coronary diseases, atherosclerosis, and cerebral infarction [12]. Several studies have demonstrated the protective effects of CP on ischemia/reperfusion-induced microvascular dysfunction including myocardial and hepatocellular injury [12-14].

However, no study has been conducted to demonstrate the effects of CP on vascular injury-related brain disease. The goal of the present study was to examine whether CP treatments ameliorate the brain damage induced by chronic cerebral hypoperfusion. CP treatments restored the myelin basic protein (MBP) degradation and microglial activation in the white matter and hippocampus of chronic BCCAo rats. The expression levels of inflammatory mediators, such as cyclooxygenase-2 (COX-2), interleukin-1 beta (IL-1β), and interleukin-6 (IL-6), were reduced in the chronic BCCAo rats given CP treatment versus those given vehicle treatments. The administration of CP mitigated altered mitogen-activated protein kinase (MAPK) signaling in the hippocampus of chronic BCCAo rats.

## Methods

### Animals

A total of 55 male Wistar rats were used in the chronic BCCAo experiment (12 weeks old; Charles River Co., Gapeung, South Korea). The rats were housed for two weeks at the beginning of the experiment in a vivarium at the Korea Institute of Oriental Medicine under controlled temperature (22 ± 1°C) and humidity (55 ± 10%) with a 12 h light/dark cycle (lights on at 08:00 h). Food and water were provided *ad libitum* to all rats throughout the experiment. The Institutional Animal Care and Use Committee of the Korea Institute of Oriental Medicine approved all protocols described in this report.

### Animal surgery and drug administration

The Wistar rats were anesthetized with 5% isoflurane in a 70% nitrous oxide and 30% oxygen mixture. Anesthesia was maintained with 3% isoflurane during the surgical procedure. A midline incision was made to expose both common carotid arteries, which were then tightly double-ligated with silk sutures. Control rats were subjected to a sham-operation that consisted of the same procedure without BCCAo. Rectal temperature was maintained at 37.0 ± 0.5°C with a heating pad throughout the surgical procedure. During hypoperfusion, two rats showed neurological symptoms, such as seizures with squatting. These animals died within one week after surgery. In addition, seven rats that lost 20% or more of their pre-surgical weight during drug or vehicle administration were excluded from the present study.

CP was obtained from Sam Chun Dang Pharm. Co., Ltd (Seoul, Korea). Each pill contained 17.5 mg of SM, 3.4 mg of PN, and 0.2 mg of DAG. The rats used in the BCCAo experiment were separated into three groups: a sham-operated group (oral administration of the drug vehicle, n = 12), a BCCAo group (oral administration of the drug vehicle, n = 17), and a BCCAo group that received daily oral administration of the drug (200 mg/kg CP, n = 17). The vehicle/drug treatment was started on the 20th day after BCCAo surgery and was continued until the 41st day after first vehicle/drug treatment (Additional file 1: Figure S1). During drug administration, the vehicle and CP treatment groups each lost four rats due to the stress related to long-term oral feeding. The CP group showed no toxicity in terms of general behavioral changes or mortality.

### Western blot analysis

After the last administration of CP or vehicle, on day 63 after BCCAo surgery (Additional file 1: Figure S1), all of the rats were decapitated, and their brains were microdissected and then frozen. Tissue homogenates of the hippocampus were prepared, and SDS-polyacrylamide gel electrophoresis (PAGE) was performed as described previously [7]. Briefly, individual tissue samples were weighed and then homogenized in ice-cold RIPA buffer containing 25 mM Tris HCl, pH 7.6, 150 mM NaCl, 1% NP-40, 1% sodium deoxycholate, 0.1% SDS (Thermo Scientific, Waltham, MA, USA), protease inhibitor cocktail solution (GenDEPOT, Barker, TX, USA), and phosphatase inhibitor cocktail solution (GenDEPOT). The homogenates were then centrifuged at 12,000 × g for 20 min at 4°C, and the supernatant was harvested, snap-frozen, and stored at −70°C until use. The protein concentration of the tissue homogenates was determined with the BCA assay (Thermo Scientific). Equal amounts of protein (40 μg) were resolved *via* SDS-PAGE and transferred to polyvinylidene difluoride (PVDF) membranes. The membranes were incubated with antibodies to p-ERK, ERK (Cell Signaling Technology, Beverly, MA, USA), p-p38 MAPK, p38 MAPK (Cell Signaling), p-JNK, JNK (Cell Signaling), COX-2 (Santa Cruz Biotechnologies, CA, USA), IL-1β (Millipore Corporation, Billerica, MA, USA), IL-6 (Abcam, San Francisco, CA, USA), and GAPDH (Santa Cruz Biotechnologies). An enhanced chemiluminescence (ECL) kit (Thermo Scientific) and a Lumino Image Analyzer (Las-4000; Fujifilm, Tokyo, Japan) were used for detection.

### Immunohistochemistry

After undergoing all of the drug administrations, the rats were euthanized by lethal overdose of Zoletil (50 mg/kg) and Rompun (5 mg/kg) followed by intracardiac perfusion with 4% paraformaldehyde. The brains were removed, postfixed, treated with distilled water containing 30% sucrose for cryoprotection, snap-frozen, and then cut into 40-μm sections on a freezing microtome. The sections were blocked overnight at 4°C with a solution of 3% casein in PBST and incubated with a series of primary antibodies for 1 h at room temperature. The following primary antibodies were used in the present study: anti-MBP (Abcam), anti-Iba-1 (Wako, Tokyo Japan), anti-OX-6 (Abcam), and anti-NeuN (Millipore Corporation). The sections were washed with PBS three times for 10 min each and then incubated with the appropriate biotinylated secondary antibodies (Thermo Scientific) for 2 h and subsequently with ExtrAvidin peroxidase conjugate (Sigma Aldrich) for 1 h. Finally, the immunostaining was visualized using the Vector SG substrate kit and the Vector DAB kit (Vector Laboratories, Burlingame, CA, USA) for peroxidase. The sections were mounted onto resin-coated slides and allowed to dry for up to one week. The dried sections on the slides were coverslipped with Permount reagent.

### Statistical analysis

One-way ANOVA was conducted to assess the effects of CP on changes in the number of OX-6 and Iba-1 positive cells as well as on the expression levels of MBP, COX-2, IL-1β, IL-6, and MAPKs induced by chronic BCCAo. Post-hoc analyses (LSD) were subsequently conducted to determine the effects of the CP treatment. P values of less than 0.05 were considered significant unless otherwise specified. All data are expressed as the means ± SEM.

## Results

### CP restored the reduced expression of MBP in the white matter and hippocampus induced by chronic BCCAo

**Figure 1:**
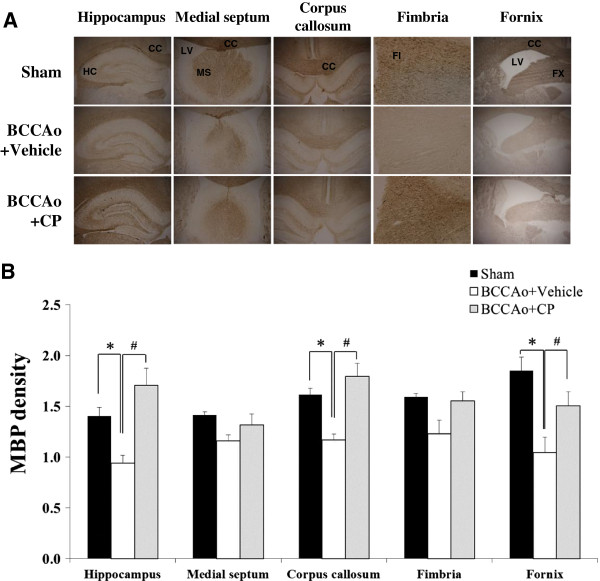
**Effects of CP on the chronic BCCAo**-**induced MBP reduction in the white matter and hippocampus.** Immunohistological staining was performed to evaluate the expression levels of MBP in the medial septum, corpus callosum, fimbria, fornix, and hippocampus in the sham-control group (n = 4), BCCAo + Vehicle group (n = 5), and BCCAo + CP group (n = 5). **(A)** Representative photomicrograph of MBP-positive cells. **(B)** MBP levels were decreased in the corpus callosum, fimbria, fornix, and hippocampus of the chronic BCCAo rats compared to control rats (*). Relative to the chronic BCCAo rats given vehicle, the chronic BCCAo-induced reduction of MBP expression in the CP-treated chronic BCCAo rats was not observed (#). CC, corpus callosum; HC, hippocampus; LV, lateral ventricle; FI, fimbria; FX, fornix.

Chronic cerebral hypoperfusion leads to lesions in the white matter through the disruption of myelin sheaths and a loss of oligodendrocytes [15]. MBP is a crucial component of the myelin sheath. We examined the effects of CP on chronic BCCAo-induced MBP breakdown in the medial septum, corpus callosum, fimbria, fornix of the white matter, and hippocampus. An ANOVA revealed significant group effects in the corpus callosum, fimbria, fornix, and hippocampus (F(2,11) ≥ 3.79, p < 0.05). Post-hoc analyses of the group effects revealed that, compared to the sham-operated control rats, the expression level of MBP in the BCCAo rats with vehicle treatment was significantly decreased in white matter regions and the hippocampus (Figure 1). Interestingly, the reduced level of MBP was not observed in the chronic BCCAo rats administered CP, implying that CP treatment could ameliorate the degradation of the myelin sheath induced by chronic cerebral hypoperfusion. An ANOVA for the medial septum showed no group effects. On the other hand, the grey matter such as cerebral cortex, which is located near CA1 region of hippocampus vulnerable to chronic cerebral hypoperfusion, did not show alteration on MBP expression (Additional file 2: Figure S2). In addition, neuronal cell death, determined by counting NeuN-positive cells (neuronal antibody), was observed in CA1, CA3, and DG subfields of the hippocampus of rats with chronic BCCAo compared to sham-operated control rats. Reversely, this neuronal cell death was declined in CP-treated chronic BCCAo rats. However, these findings did not show statistical significance (Additional file 3: Figure S3).

### CP inhibited microglial activation in the white matter and hippocampus induced by chronic BCCAo

**Figure 2:**
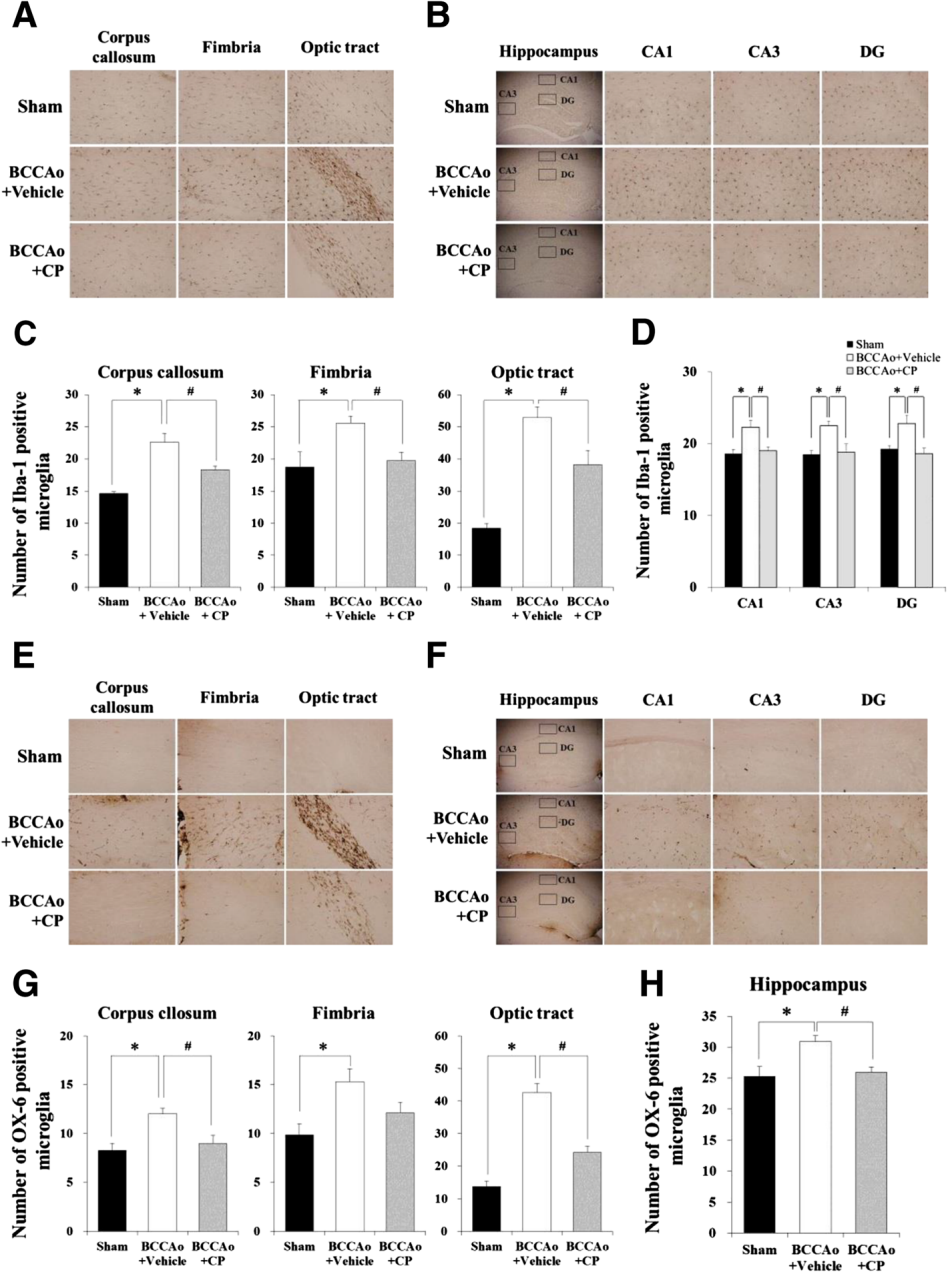
**Effects of CP on the chronic BCCAo**-**induced microglial activation in the white matter and hippocampus.** Immunohistological staining was conducted to investigate the expression of Iba-1 and OX-6 in regions of the white matter and the hippocampal subregions (CA1, CA3, and DG), in the sham-control group (n = 4), BCCAo + Vehicle group (n = 5), and BCCAo + CP group (n = 5). **(A)** Representative photomicrograph of Iba-1 **(A** and **B)** and OX-6 **(E** and **F)** positive cells. **(B)** The number of Iba-1 **(C** and **D)** and OX-6 **(G** and **H)** positive cells was increased in the chronic BCCAo rats compared to the sham-control rats (*), an effect that was not observed in the chronic BCCAo rats under CP administration (#).

To investigate the effect of CP on neuroinflammation induced by chronic cerebral hypoperfusion, the present experiment measured the number of Iba-1-positive cells, the resident microglial cells in the brain, in identical sections of the corpus callosum, fimbria, optic tract, and hippocampal subregions (CA1, CA3, and DG) to examine the effects of CP on BCCAo-induced microglial activation. An ANOVA revealed significant group effects of Iba-1-positive cells in the corpus callosum, fimbria, and hippocampal subregions (CA1, CA3, and DG) (F(2,11) ≥ 6.04, p < 0.01). Post-hoc analyses of the group effects revealed that the number of Iba-1-positive cells in the vehicle-treated BCCAo rats was significantly higher than the sham-control rats in all of the regions examined (Figure 2A through D). CP administration reduced the increase in Iba-1-positive cells induced by BCCAo in all of the brain regions examined.

In addition, we measured the number of OX-6-positive cells, which are activated microglial cells, in the corpus callosum, fimbria, and optic tract. An ANOVA revealed significant group effects for OX-6-positive cells in the corpus callosum, fimbria, and optic tract (F(2,11) ≥ 5.21, p < 0.05). Post-hoc analyses of the group effects revealed that the number of OX-6-positive cells in the vehicle-treated BCCAo rats was significantly higher than in the sham-control rats in the examined brain regions (Figure 2E through H). The BCCAo rats administered CP showed a reduction in OX-6-positive cells compared to the BCCAo rats given the vehicle. The Iba-1 and OX-6 results suggest that CP treatments mitigated chronic BCCAo-induced microglial activation in the white matter and hippocampus.

### CP attenuated the increase of COX-2, IL-6, and IL-1β expression in the hippocampus of rat with chronic BCCAo

**Figure 3:**
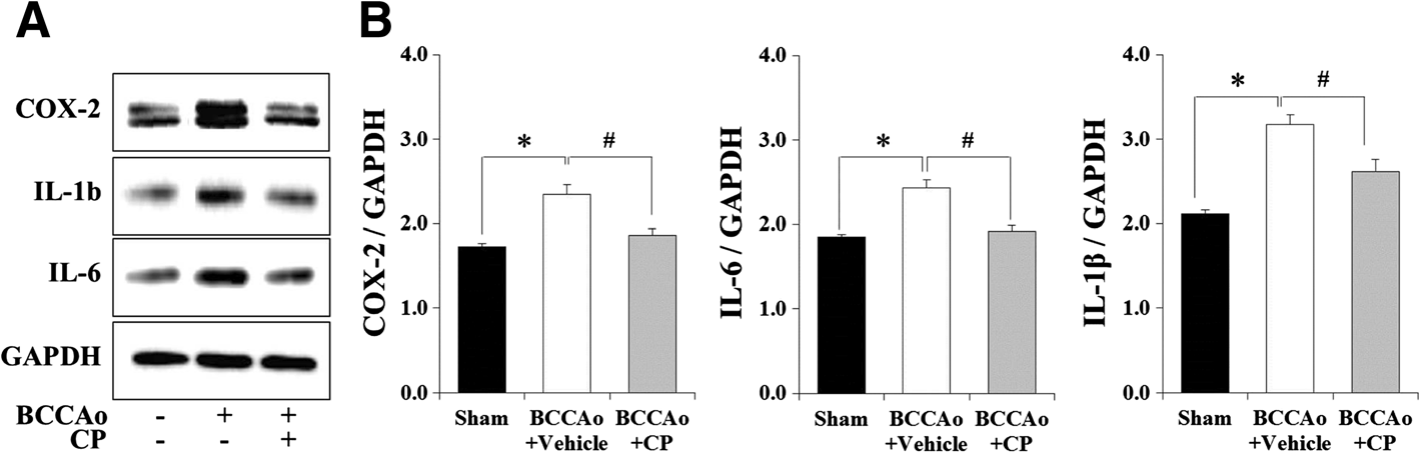
**Effects of CP on hippocampal levels of inflammatory mediators in chronic BCCAo rats. (A)** Representative western blot of COX-2, IL-1β, and IL-6. **(B)** The levels of inflammatory mediators (COX-2, IL-1β, and IL-6) were increased in the BCCAo + Vehicle group (n = 8), compared to the sham-control group (n = 8,*). CP administration (n = 8) attenuated the increased levels of hippocampal inflammatory mediators induced by chronic BCCAo (#).

Activated microglial cells release a variety of proinflammatory cytokines, eventually leading to neuronal injury [16]. Therefore, to examine the effects of CP on BCCAo-induced inflammation, we used western blotting to measure hippocampal levels of COX-2, IL-1β, and IL-6. An ANOVA revealed significant group effects of the measured proinflammatory markers (F(2,21) ≥ 15.65, p < 0.001). Post-hoc analyses of the group effects revealed that chronic BCCAo increased the levels of these proinflammatory markers in the hippocampus (Figure 3A and B). The increase in proinflammatory markers by chronic BCCAo did not occur in the chronic BCCAo rats treated with CP (Figure 3A and B).

### CP blocked the hyperphosphorylation of MAPKs in the hippocampus of rats with chronic BCCAo

**Figure 4:**
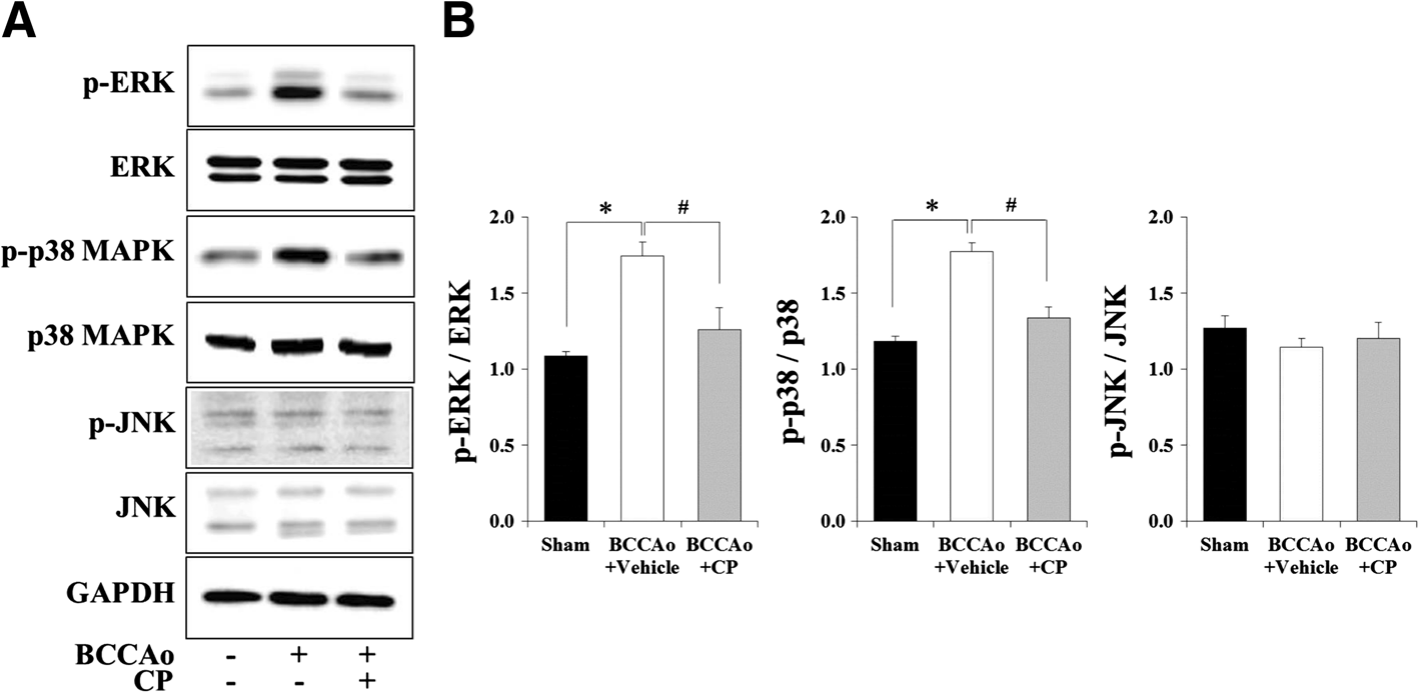
**Effects of CP on increased levels of hippocampal phosphorylated MAPKs induced by chronic BCCAo. (A)** Representative western blot of total MAPKs and phosphorylated MAPKs. **(B)** The hippocampal levels of phosphorylated ERK (p-ERK) and p38 (p-p38), but not JNK (p-JNK), were increased by chronic BCCAo (*). CP administration attenuated the increased levels of hippocampal p-ERK and p-p38 induced by chronic BCCAo (#). Each group had 8 subjects.

To determine the effects of CP on the levels of phosphorylated MAPKs induced by chronic BCCAo, we used western blotting to measure the hippocampal levels of phosphorylated MAPKs, such as ERK, p38 MAPK, and JNK. An ANOVA of phosphorylated ERK and p38, but not of JNK, showed significant group effects (F(2,21) ≥ 16.05, p < 0.001). An ANOVA of total ERK, p38, and JNK showed no group effects. Subsequent post-hoc analyses showed that the levels of phosphorylated ERK and p38 were increased in the hippocampus of chronic BCCAo rats treated with vehicle compared to the sham-control rats (Figure 4A and B). Increased hippocampal levels of phosphorylated ERK and p38 MAPKs induced by chronic BCCAo were not observed in the BCCAo rats under CP administration (Figure 4A and B). These results indicate that CP may possess anti-inflammatory properties through the inhibition of MAPK signaling.

## Discussion

A number of studies have demonstrated the beneficial properties of CP in gut ischemia-reperfusion (I/R)-induced hepatic microvascular dysfunction and hepatocellular injury [12], thrombus development [17], I/R-induced microcirculatory disturbance [14], and myocardial fibrosis [13]. Despite the widespread use of CP for the treatment of cardiovascular and cerebrovascular diseases, no study has examined the action of CP upon white matter and hippocampal damage by chronic BCCAo. The present study demonstrates the protective effect of CP against chronic BCCAo-induced brain damage by restoring white matter lesions and altering the inflammatory response. We previously reported that chronic cerebral hypoperfusion induced by chronic BCCAo results in cognitive impairments, microglial activation in the white matter, and phosphorylation of hippocampal MAPKs. Furthermore, we showed that *Fructus mume* extract improves the cognitive impairment induced by chronic cerebral hypoperfusion [7]. Our previous studies and the present study indicate that the BCCAo animal model is a well-established model for examining the efficacies of drug candidates for the treatment of VaD. The white matter constitutes approximately 50% of the human brain volume, and the hippocampus plays an important role in memory formation, organization, and storage, which these regions are highly vulnerable to chronic cerebral hypoperfusion [18]. Long-term changes in regional cerebral blood flow and glucose utilization in the white matter, including the corpus callosum, internal capsule, and optic tract, of chronic BCCAo rats have been reported [19], and it is subsequently involved in development of cognitive decline by damage of the white matter [20,21]. A recent study demonstrated that white matter and hippocampal damage are induced by the degradation of the myelin sheath, a component of the white matter and hippocampus [15]. MBP is involved in the stability of the myelin sheath [15]. Therefore, MBP expression levels indicate the severity of white matter and hippocampal damage. The present study showed that MBP expression was decreased in the white matter and hippocampus under chronic BCCAo, but the decrease in MBP expression was not observed in the BCCAo rats given CP treatments. In addition, MBP expression did not reduced in cerebral cortex, one of the grey matter regions (Additional file 2: Figure S2). These results are supported by the finding that neuronal damage was induced predominantly in white matter rather than the grey matter in moderate cerebral hypoperfusion animal [22]. Furthermore, CP treatment decreases neuronal cell death in the hippocampus induced by chronic BCCAo. These findings suggest that CP may exert protective effects against white matter and hippocampal damage induced by chronic cerebral hypoperfusion *via* inhibition of myelin degradation and the neuronal death.

Inflammation plays a key role in white matter lesions induced by chronic cerebral hypoperfusion [23,24]. Based on evidence of microglial activation and increased expression of proinflammatory mediators in chronic BCCAo rats, neuroinflammation is associated with the induction of white matter lesions following chronic cerebral hypoperfusion [10,25,26]. Microglia are a major source of proinflammatory cytokines, such as tumor necrosis factor-α (TNF-α) and IL-1β [16]. These cytokines initiate intracellular signaling cascades, including MAPK and nuclear factor-kappa B (NF-*k*B), by interacting with their immune cell receptor, and subsequently exacerbate myelin degradation [27,28]. MAPK signaling plays a major role in synaptic plasticity and hippocampus-dependent memory [29] and is involved in microglial activation and the production of proinflammatory mediators [27]. Actually, hyperphosphorylation of MAPKs and overproduction of proinflammatory cytokines, such as IL-1β [8] and IL-6 [30], is observed in the brains of rats under chronic BCCAo [7,31]. In addition, MAPK signaling is involved in the upregulation of proinflammatory cytokines, including TNF-α, IL-1β, and IL-6, in the hippocampus and microglia. Blockade of MAPK signaling using a specific inhibitor resulted in a reduction of the increased expression of proinflammatory cytokines induced by a stimulator [32,33]. Therefore, many studies have suggested that the inhibition of microglial activation and inflammatory responses might contribute to the prevention of white matter and hippocampal lesions [20,34]. The present study showed that CP inhibits MBP breakdown, microglial activation, the upregulation of proinflammatory mediators, and the hyperphosphorylation of MAPKs in the white matter and hippocampus of rats under chronic BCCAo.

## Conclusion

We demonstrate that CP ameliorates white matter and hippocampal damage induced *via* chronic cerebral hypoperfusion by attenuating the induction of white matter lesions and the neuroinflammatory response. Furthermore, the suppression of the inflammatory response by CP might be involved in the downregulation of MAPK signaling. These findings suggest that CP may be a potential candidate for the prevention and treatment of AD and VaD.

## Competing interests

All authors declared that they have no competing interests.

## Authors’ contributions

KML and WKJ designed the study and wrote the manuscript. JHB and ISL performed the experimentation. JSH, BYK, and HWK carried out the statistical analysis and interpretation of data. All authors read and approved the final manuscript.

## Pre-publication history

The pre-publication history for this paper can be accessed here:

http://www.biomedcentral.com/1472-6882/13/334/prepub

## Supplementary Material

Additional file 1: Figure S1 Experimental design. Click here for file

Additional file 2: Figure S2 Effect of CP on damage of the grey matter by chronic BCCAo. Immunohistological staining was performed to assess the expression levels of MBP in cerebral cortex of grey matter in the sham-control group (n=4), BCCAo+Vehicle group (n=6), and BCCAo+CP group (n=7). (A) Representative photomicrograph of MBP-positive cells. (B) Unlike apparent difference of MBP level in the hippocampus, MBP levels in the cerebral cortex, an adjacent grey matter region to the hippocampus, show no significant difference among the groups. CC, cerebral cortex; CA 1, cornu ammonis 1. Click here for file

Additional file 3: Figure S3 Effect of CP on the chronic BCCAo-induced neuronal cell reduction in the hippocampus. Immunohistological staining was accomplished to evaluate the NeuN-positive cells (neuronal antibody) in CA1, CA3, and DG subfields of hippocampus in the sham-control group (n=4), BCCAo+Vehicle group (n=6), and BCCAo+CP group (n=7). (A) Representative photomicrograph of NeuN-positive cells. (B) NeuN-positive cells were decreased in CA1, CA3, and DG subfields of the chronic BCCAo rats compared to sham-operated control rats. Relative to the chronic BCCAo rats given vehicle, the reduction of MBP expression in the CP-treated chronic BCCAo rats was not observed. The statistical significances among these results were not observed. CA 1 and 3, cornu ammonis 1 and 3; DG, dentate gyrus. Click here for file
