# Supplementary figures and images for: Cardiotonic pill attenuates white matter and hippocampal damage via inhibiting microglial activation and downregulating ERK and p38 MAPK signaling in chronic cerebral hypoperfused rat
Source: BMC Complement Altern Med. 2013 Nov 26;13:334. doi: 10.1186/1472-6882-13-334 (PMC4222777; doi:10.1186/1472-6882-13-334)

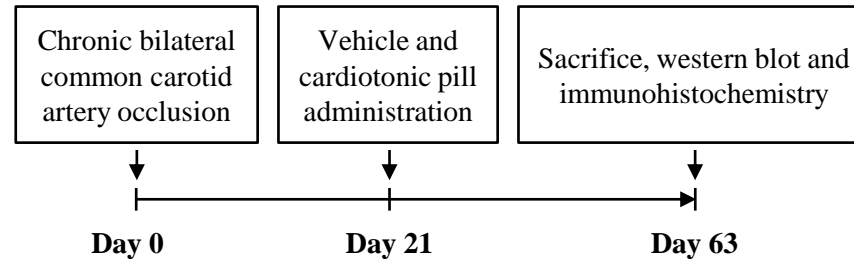

Supplement: Additional file 1: Figure S1 — Experimental design. [file 1472-6882-13-334-S1.pdf]

**A**

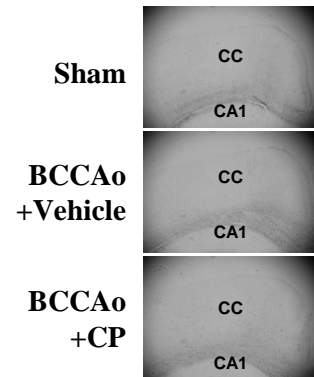

**B**

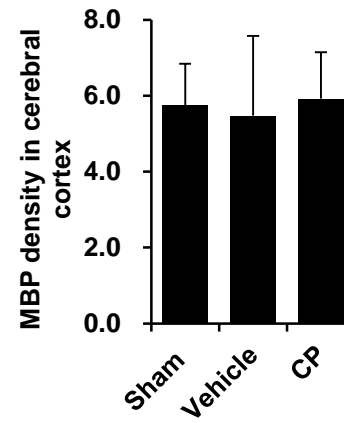

Supplement: Additional file 2: Figure S2 — Effect of CP on damage of the grey matter by chronic BCCAo. Immunohistological staining was performed to assess the expression levels of MBP in cerebral cortex of grey matter in the sham-control group (n=4), BCCAo+Vehicle group (n=6), and BCCAo+CP group (n=7). (A) Representative photomicrograph of MBP-positive cells. (B) Unlike apparent difference of MBP level in the hippocampus, MBP levels in the cerebral cortex, an adjacent grey matter region to the hippocampus, show no significant difference among the groups. CC, cerebral cortex; CA 1, cornu ammonis 1. [file 1472-6882-13-334-S2.pdf]

**A**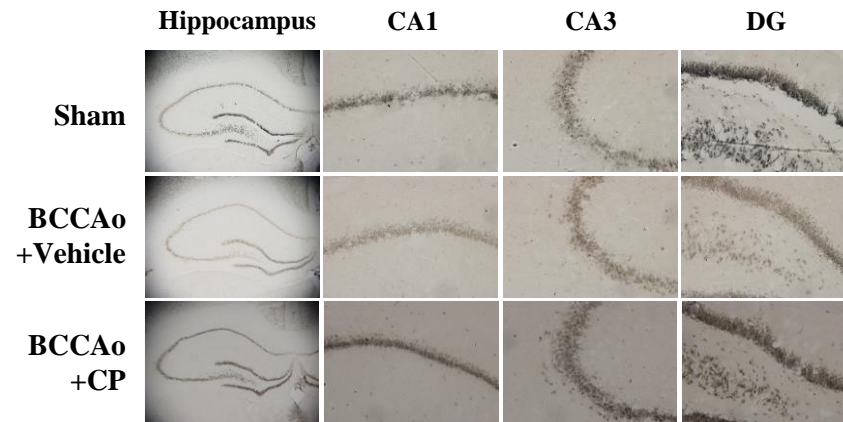**B**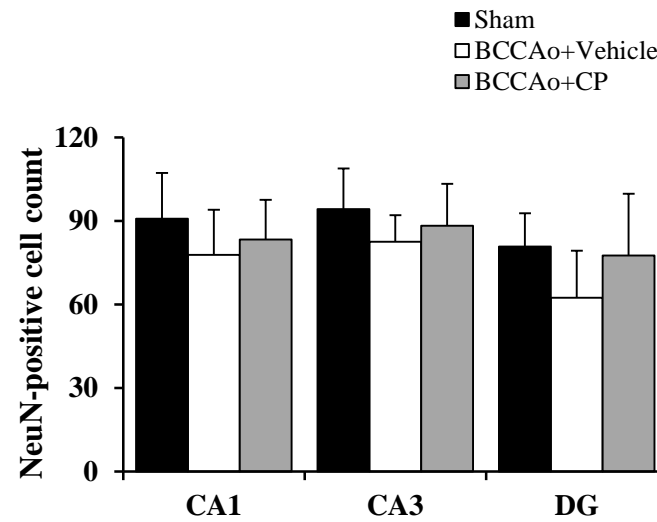

Supplement: Additional file 3: Figure S3 — Effect of CP on the chronic BCCAo-induced neuronal cell reduction in the hippocampus. Immunohistological staining was accomplished to evaluate the NeuN-positive cells (neuronal antibody) in CA1, CA3, and DG subfields of hippocampus in the sham-control group (n=4), BCCAo+Vehicle group (n=6), and BCCAo+CP group (n=7). (A) Representative photomicrograph of NeuN-positive cells. (B) NeuN-positive cells were decreased in CA1, CA3, and DG subfields of the chronic BCCAo rats compared to sham-operated control rats. Relative to the chronic BCCAo rats given vehicle, the reduction of MBP expression in the CP-treated chronic BCCAo rats was not observed. The statistical significances among these results were not observed. CA 1 and 3, cornu ammonis 1 and 3; DG, dentate gyrus. [file 1472-6882-13-334-S3.pdf]
